# A two-way street – cellular metabolism and myofibroblast contraction

**DOI:** 10.1038/s41536-024-00359-x

**Published:** 2024-04-03

**Authors:** Anne Noom, Birgit Sawitzki, Petra Knaus, Georg N. Duda

**Affiliations:** 1 Julius Wolff Institute (JWI), Berlin Institute of Health and Center for Musculoskeletal Surgery at Charité – Universitätsmedizin Berlin, 13353 Berlin, Germany; 2 BIH Center for Regenerative Therapies (BCRT), Berlin Institute of Health at Charité – Universitätsmedizin Berlin, 13353 Berlin, Germany; 3 Department of Infectious Diseases and Respiratory Medicine, Charité – Universitätsmedizin Berlin, Corporate Member of Freie Universität Berlin and Humboldt University of Berlin, 13353 Berlin, Germany; 4 Center of Immunomics, Berlin Institute of Health at Charité – Universitätsmedizin Berlin, 13353 Berlin, Germany; 5 Institute of Chemistry and Biochemistry – Biochemistry, Freie Universität Berlin, 14195 Berlin, Germany

**Keywords:** Stem-cell research, Regenerative medicine, Stem-cell niche

## Abstract

Tissue fibrosis is characterised by the high-energy consumption associated with myofibroblast contraction. Although myofibroblast contraction relies on ATP production, the role of cellular metabolism in myofibroblast contraction has not yet been elucidated. Studies have so far only focused on myofibroblast contraction regulators, such as integrin receptors, TGF-β and their shared transcription factor YAP/TAZ, in a fibroblast-myofibroblast transition setting. Additionally, the influence of the regulators on metabolism and vice versa have been described in this context. However, this has so far not yet been connected to myofibroblast contraction. This review focuses on the known and unknown of how cellular metabolism influences the processes leading to myofibroblast contraction and vice versa. We elucidate the signalling cascades responsible for myofibroblast contraction by looking at FMT regulators, mechanical cues, biochemical signalling, ECM properties and how they can influence and be influenced by cellular metabolism. By reviewing the existing knowledge on the link between cellular metabolism and the regulation of myofibroblast contraction, we aim to pinpoint gaps of knowledge and eventually help identify potential research targets to identify strategies that would allow switching tissue fibrosis towards tissue regeneration.

## Introduction

Tissue fibrosis is the overgrowth, hardening and/or scarring of tissues, which is often paired with excessive and dysregulated deposition of extracellular matrix (ECM)^1^. Although fibrosis closes an injury gap, it is often negatively associated with a lack of tissue regeneration or failure to repair and restore damaged tissues^2^. Cardiac, lung and liver fibrosis are, for instance, responsible for 45 percent of all mortalities in the United States^1,3,4^. Therefore, understanding the mechanisms underlying tissue fibrosis would allow to identify potential targets for a de-railed regeneration and avoid scarring in wound healing.

One mechanism considered to be common across organs suffering from fibrotic changes is the so-called fibroblast-myofibroblast transition (FMT). Fibroblasts are stromal cells that help in the repair of damaged tissues in various ways. They are thought to get activated and transition to myofibroblasts by mechanical strain signals^5^ and the anti-inflammatory cytokine transforming growth factor-beta (TGF-β)^2,6,7^. TGF-β1 is best described for this function since, upon activation, it signals through the Smad2/3 pathway that results in the upregulation of Smad target genes including many ECM molecules and integrin receptors. Integrin receptors can pull on the arginylglycylaspartic acid (RGD) peptide of the TGF-β latent complex to make a cleavage site available for the release of TGF-β^8,9^, thereby inducing a feed-forward loop supporting the process once it has started with the site-specific release of active TGF-β. This shows the importance of TGF-β in tissue fibrosis, and many have tried to target them for attenuating fibrosis. Indeed, inhibiting FMT by blocking TGF-β1 by CAT-192^10^ or by inducing myofibroblast apoptosis^11^ by the single chain antibody C1-3 ameliorated fibrosis in multiple organs. Although promising, no effective drugs are currently in clinical use^12^. Thus, a better understanding of FMT in the different organ settings and identifying underlying mechanisms that are common across appears to provide the necessary knowledge that precedes any targeted therapy development.

**Fig. 1:**
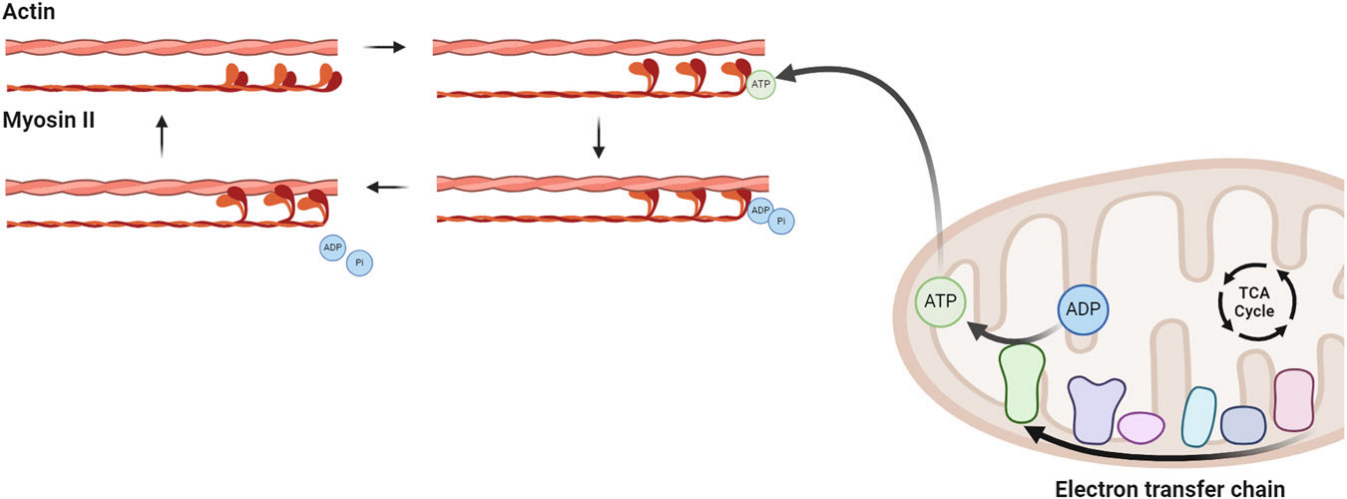
Working mechanism behind myofibroblast contraction. Adenosine triphosphate (ATP) is produced by the tricarboxylic acid (TCA) cycle and electron transfer chain and binds to an unattached myosin head. Here, it is hydrolysed and causes the myosin head to form a cross-bridge. Adenosine diphosphate (ADP) and inorganic phosphate (Pi) release causes the myosin head to change position and the actin filament to thereby move causing myofibroblast contraction. Created with BioRender.com.

Another common mechanism across fibrotic organs caused by FMT is myofibroblast contraction. Myofibroblast contraction is an essential process during tissue regeneration that not only closes the wound but also restores the intrinsic tissue and matrix tension^13^. Cell contraction enables ECM contraction through the sliding of non-muscle myosin II (NMMII) along actin stress fibres (Fig. 1). NMMII binds to actin upon adenosine triphosphate (ATP) hydrolysis forming a so-called cross bridge. Once adenosine diphosphate (ADP) and inorganic phosphate (Pi) are released from myosin, the actin filament is pulled across myosin which results in cellular contraction^14^. Such cellular contraction pulls on ECM components thus forming a more compact ECM network and eventually enables wound closure. However, in fibrosis, myofibroblasts perform contractions that are frequently called excessive and may result in hypertrophic scarring^13^. Inhibition of myosin by chemical inhibitor Ble has been shown to attenuate liver fibrosis in vivo^7^, but also here no effective drugs targeting myosin for fibrosis are in clinical use yet. Hence, other mechanisms might play a role in myofibroblast contraction and could be considered when targeting a therapeutic approach.

Since myofibroblast contraction is a strong energy-consuming process involving high amount of ATP hydrolysis, it suggests to be linked to cellular metabolism during FMT. Cellular metabolism is a map full of interlinked pathways, such as glycolysis and the tricarboxylic acid (TCA) cycle, which make up the sum of biochemical processes that either produce or consume energy and macromolecules. Glycolysis is, for instance, the conversion of glucose to pyruvate. Pyruvate is then shuttled into the TCA cycle to produce ATP through oxidative phosphorylation^15–17^. However, cancer cells have shown a different ATP production via the so-called Warburg effect. This includes the fermentation of lactate, even in the presence of oxygen, and is characterised by high glycolytic rates^15,18^. Although described for cancer cells, stromal cells have shown similarities to the Warburg effect during migration and proliferation. Myofibroblasts convert pyruvate to lactate for ATP production^19,20^ and thereby resemble another cancer characteristic, namely enhanced glutaminolysis^21–23^. Glutaminolysis is the conversion of glutamine to glutamate, which can then be used for multiple pathways, but is often used to replenish the TCA cycle. In oncology, the knowledge about metabolic dependencies for cancer cells has led to novel drug development that targets metabolic vulnerabilities^24^. However, this has not yet been transferred to treatment strategies for fibrotic diseases.

More recently, energy metabolism has appeared as one of the processes that is regulated by mechanical cures (extensively reviewed by Romani et al.^25^). However, the exact mechanisms and receptors involved in tissue contraction, especially how this is regulated by energy metabolism, have not been fully elucidated. This review addresses how relevant cellular metabolism is for tissue contraction.

Within this review we aim to discuss the knowledge available linking FMT regulators, ECM properties and energy metabolism. Additionally, we would like to compare the mechanisms between mechanical cues and biochemical signalling that led to altered energy metabolism during FMT. We further highlight mechanisms eventually common across multiple organs and describe how these mechanisms may be leveraged for the design of future studies to examine potential targets that lead to the switch between regeneration and aberrant fibrosis.

## Integrin receptors influence cellular metabolism in myofibroblast contraction through ECM sensing

Myofibroblast contraction is essential to wound healing but should be tightly regulated during tissue regeneration to prevent scarring. The contraction is not only essential for wound closure but also restores the intrinsic tension that was initially lost by injury of the organ. When dysregulated, persistent myofibroblast contraction causes tissue fibrosis^13^. Although cellular metabolism plays a role in myofibroblast contractility, the influence of cellular metabolism on myofibroblast contraction has surprisingly not been elucidated. The few studies have only focused on actin filament dynamics and apparently did not consider the cell/tissue contractility. Since myofibroblast contraction can be regulated by integrin receptors, we will here focus on the influence of cellular metabolism on the integrin regulation of myofibroblast contraction.

### ECM properties regulate myofibroblast contraction

Myofibroblast contraction can be regulated by integrin receptors. Integrins are single transmembrane-spanning receptors which bind at their extracellular site to ECM components, such as fibronectin (FN) and collagen, while at the cytosolic domain, they activate signalling cascades by recruiting adaptor proteins Talin and Kindlin. Interestingly, integrins can signal either inside-out or outside-in, depending on ligand binding. The inside-out signalling is the binding of actin to integrin receptors facilitated by Talin and Kindlin that activates the receptor from the cytosolic side, causing it to reach out and grab onto FN or collagen^26^. Similarly, binding to the RGD sequence in FN can switch on the intracellular signalling cascade by activating focal adhesion kinases that associate with the C-terminal tail of integrins, referred to as outside-in signalling^27,28^.

Additionally, integrins respond differently to soft and stiff ECM properties. First, soft ECM defined with Young’s modulus (*E*) around 2 kPa^29^, such as FN, is mainly bound by αv integrins, whereas stiff ECM defined with *E* above 35 kPa^29^, like collagen, is bound by β1 integrins^30^. This leads to different signalling cascades, such as the Rho-mDia axis and the Rho-Rock axis, respectively. The Rho-mDia axis is responsible for the assembly of actin filaments and is often paired with the migration of fibroblasts, while the Rho-Rock axis is necessary for myosin II transcription and could therefore cause the contraction^30^. Indeed, blocking myosin II by RNA interference was necessary to prevent the contraction observed in tissue fibrosis^31^. Thus, integrins have a dual role in the assembly of stress fibres but need stiff ECM to actually promote myofibroblast contraction.

### ECM properties activate alternative metabolic pathways

**Fig. 2:**
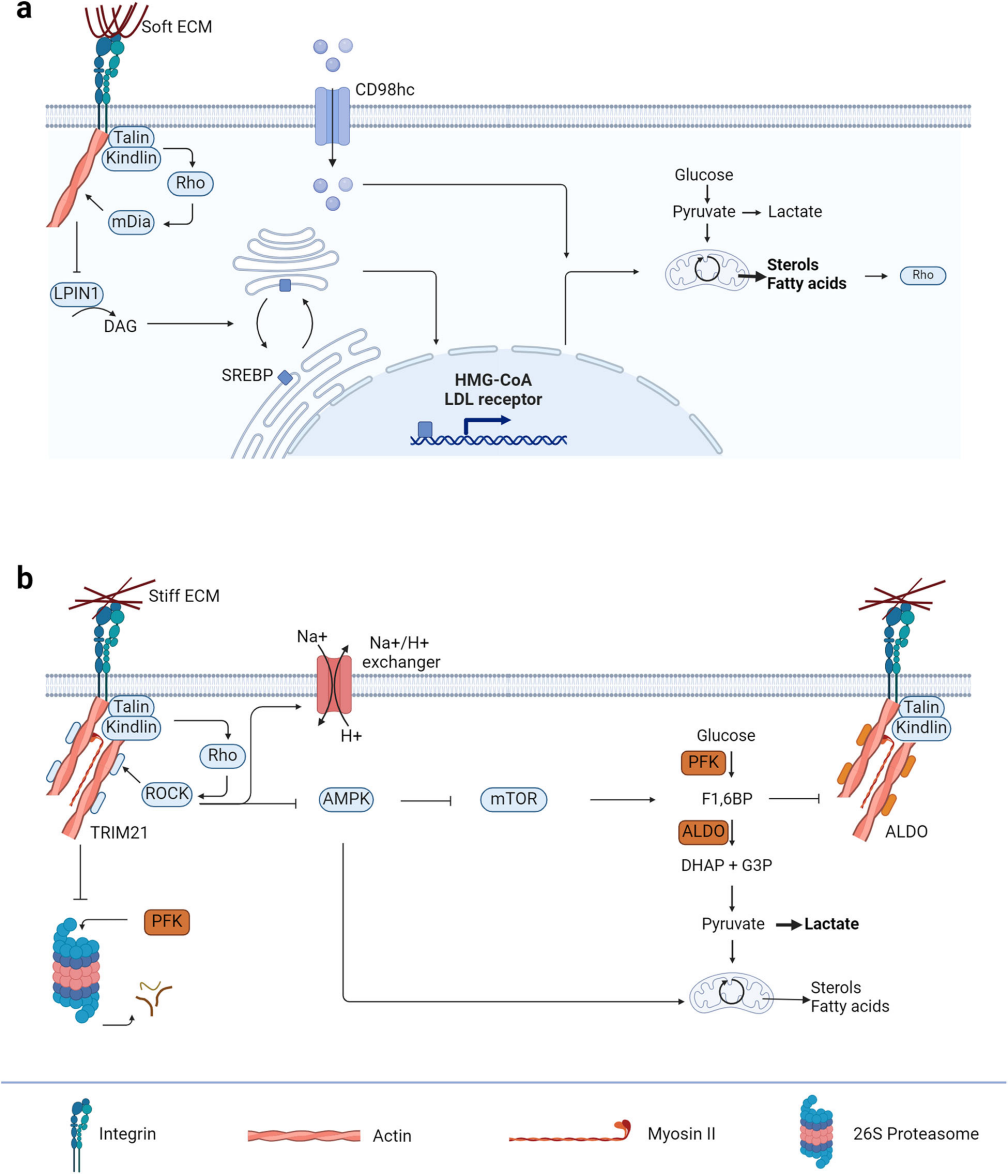
Integrin receptors influence cellular metabolism through ECM sensing. **a** Soft ECM activates the Rho-mDia signalling cascade leading to increased lipid synthesis by accumulating SREBP at the Golgi apparatus that in turn after being cleaved can upregulate the expression of HMG-CoA and LDL receptor for the mevalonate and lipid synthesis pathway. Additionally, soft ECM regulates the integrin co-receptor CD98hc to import sphingolipids which helps in increasing the lipid content in the cell and therefore also the Rho recruitment to the cell membrane. **b** Stiff ECM activates the Rho-ROCK signalling cascade leading to higher glycolytic rates by on one hand inhibiting the degradation of phosphofructokinase (PFK) and on the other hand inhibiting AMP-activated protein kinase (AMPK) and stimulating Na+/H+ exchangers to promote higher pH for elevated glycolytic flux. ALDO aldolase, DAG diacylglycerol, DHAP dihydroxyacetone phosphate, F1,6BP fructose 1,6-bisphosphate, G3P glyceraldehyde 3-phosphate, HMG-CoA β-hydroxy β-methylglutaryl-CoA, LDL low-density lipoprotein, LPIN1 phosphatidate phosphatase lipin 1, mTOR mammalian target of rapamycin. Created with BioRender.com.

As contraction needs ATP hydrolysis, it can be expected that soft and stiff ECM activate alternative metabolic pathways. Indeed, soft ECM promotes cholesterol and neutral lipid synthesis (Fig. 2a). The reduced amount of stress fibres inhibits phosphatidate phosphatase lipin 1 (LPIN1) activity, which in turn decreases diacylglycerol (DAG) levels in ER/Golgi membranes^32,33^. Consequently, sterol regulatory element-binding protein (SREBP) proteins accumulate at the Golgi apparatus where they are cleaved by proteases. The cytoplasmic domain of SREBP is thereby released and shuttles into the nucleus where it drives the expression of fatty acids and cholesterol biosynthetic enzymes, such as fatty acid synthase, β-hydroxy β-methylglutaryl-CoA (HMG-CoA) reductase, and the low-density lipoprotein (LDL) receptor^32–35^. In contrast, stiff ECM sustains glycolysis (Fig. 2b). Increased numbers of stress fibres keep tripartite motif containing-21 (TRIM21) locked from its phosphofructokinase (PFK) degradation function^36^. Hence, glycolysis gets promoted. Additionally, stiff ECM activates Rock protein, which subsequently inhibits adenosine monophosphate (AMP)-activated protein kinase (AMPK)^37^, affecting glycolysis via mammalian target of rapamycin (mTOR). Moreover, Rock promotes the activity of Na/H exchanger since high glycolytic rates lead to intracellular accumulation of lactate and acidification of cells, which negatively regulates enzymes and focal adhesions at integrins^38,39^. In summary, stiff ECM seems to promote fast ATP generation compared to soft ECM, which aids contraction.

Furthermore, soft and stiff ECM might have different effects on the mitochondria. Mitochondria are extremely dynamic organelles, constantly undergoing processes of fission and fusion depending on the cellular needs. Mitochondrial GTPases Mitofusin-1, Mitofusin-2 and Optic atrophy mediate fusion, whereas dynamin-related protein 1 (DRP1) executes fission, resulting in aerobic glycolysis. The latter is also the most mechanosensitive mitochondrial GTPase^40^. Stromal cells grown on soft ECM (*E* = 0.35 kPa) showed fragmented mitochondria, whereas stiff ECM (*E* = 40 kPa) promoted mitochondrial fusion, oxidative phosphorylation and suppressed DRP1 expression^40^. Additionally, elevated levels of DRP1 can be found in cardiac^41^, liver^42^, pulmonary and renal^40^ fibrotic tissues, relatively “soft” tissues, which implies that the in vitro situation is mimicking the in vivo environment for tissue fibrosis. In summary, soft ECM promotes aerobic glycolysis, thus slowing ATP generation, whereas stiff ECM promotes oxidative phosphorylation, thus increasing the ATP generation in stromal cells and aiding the contraction.

Added to the direct influence of ECM, integrins do also signal through biochemical signalling cascades. Two of them are already mentioned in the regulation section and seem to influence the metabolism as well. The Rho-Rock axis inhibits AMPK^37^ and therefore also the homoeostatic fatty acid oxidation (FAO) that can be observed in the heart, lungs and skin. AMPK is known to regulate catabolic metabolism and inhibit anabolic metabolism. Conversely, mTOR promotes anabolic metabolism and seems to be upregulated by the inhibition of AMPK. Moreover, integrins are known to bind focal adhesion kinases and so Src family kinases, which can also promote mTOR signalling through the phosphoinositide 3-kinase (PI3K)-Akt pathway. This signalling cascade is associated with high glycolytic rates and fast ATP production which may help in facilitating myofibroblast contraction.

### Cellular metabolism impacts integrin receptor signalling

Since metabolism supplies not only ATP but also building blocks for every aspect of cell biology, including stress fibres and ECM, energy generation should influence integrin signalling by cytoskeleton remodelling. Although not known for contractility, migration studies have shown that the ATP-to-ADP ratio increases when fibroblasts are migrating to a denser collagen matrix^43^. This increase in ATP concentration has been shown to be essential for migration and suggests a role for mitochondria. The fragmented mitochondria are found at the cell cortex and thereby seem to fuel the actomyosin cytoskeleton required for migration.

Moreover, glycolytic enzymes seem to have a role in stabilising F-actin structures. Aldolase can bind to actin, especially in stress fibres, and act as a bundling protein. By binding to actin, aldolase inhibits cell motility and spreading and thereby is not accessible for its role in glycolysis^44,45^. The latter is regulated by the abundance of glycolytic intermediates^44–46^, suggesting a potential influence of metabolism on actin dynamics.

A different way by which integrin signalling is regulated is membrane fluidity. Membrane fluidity is the property of the cell membrane that allows it to adapt its shape and movement to different conditions. Three factors influence membrane fluidity: temperature, cholesterol and type of fatty acids, where unsaturated fatty acids cause more membrane fluidity and saturated fatty acids promote more rigid membranes^47^. Cholesterol-treated fibroblasts showed increased Talin and integrin expression, indicating that higher membrane fluidity enhances integrin signalling^48,49^. Intriguingly, cellular metabolism can regulate integrin mechano-sensing via a sphingolipid metabolic pathway that is controlled by the amino acid transporter and integrin co-receptor CD98hc. Loss of CD98hc decreased sphingolipid availability and prevented proper membrane recruitment and activation of regulators of Rho^50^. Since sphingolipids are fatty acids and thus also important to membrane fluidity, this complements the hypothesis that membrane fluidity is important for integrin signalling.

To summarise this section, integrin receptors influence cellular metabolism in myofibroblast contraction through ECM sensing. First, soft ECM promotes the assembly of actin filaments, cell migration, mitochondrial fission, and fatty acid synthesis. The latter is important for membrane fluidity, which was shown to be important for enhancing integrin signalling. Second, stiff ECM promotes Rho-Rock signalling which is essential for myosin II transcription. It sustains glycolysis for fast ATP generation and if Young’s modulus gets high enough could provide a potential negative feedback loop. Thus, soft ECM seems to prime the fibroblast for its needs for myofibroblast contractions, whereas stiff ECM simply sustains those needs.

## TGF-β influences cellular metabolism in myofibroblast contraction

Although TGF-β is the best-described regulator for FMT and therefore also myofibroblast contraction, it still can be interesting to look at the influence of TGF-β on cellular metabolism and conversely the influence of cellular metabolism on TGF-β signalling. By comparing the signalling of TGF-β with integrins, we could select key pathways necessary for the contraction and the key influence on contraction. The following subsection will describe how TGF-β regulates myofibroblast contraction before going in-depth into how its signalling cascade is influenced by cellular metabolism.

### TGF-β influences myofibroblast contraction by enhancing αSMA expression, but not myosin II expression

There are three mammalian members of TGF-β, TGF-β1, TGF-β2 and TGF-β3, resembling a subgroup of the large TGF-β/bone morphogenetic protein (BMP) growth factor family. TGF-β is secreted and deposited in the ECM as a large latent complex^51^. Integrin αvβ5 and αvβ7 can bind to the RGD sequence of the complex and release mature TGF-β into the microenvironment by pulling on the latent complex to make a proteolytic cleavage site accessible for cleavage. Only after release the mature homodimeric ligand binds to their respective receptors on the cell membrane^8,9^. Their receptors are transmembrane proteins and include a type I receptor (TβRI), also known as activin receptor-like kinase 5 (ALK5)^52^, and a type II receptor (TβRII), each carrying a serine/threonine kinase domain. Upon stimulation, TβRII phosphorylates TβRI and consequently TβRI can phosphorylate Smad in the canonical signalling and mitogen-activated protein kinase (MAPK), Rho and PI3K-Akt in the non-canonical signalling^51^. The canonical signalling is well known for enhancing α-smooth muscle actin (αSMA) expression and thereby causing FMT (extensively reviewed for renal^53^, hepatic^54^, lung^55^ and cardiac fibrosis^56^)^57^. In addition, PI3K-Akt can also enhance αSMA expression when stimulated by focal adhesion kinases^58^. The inhibition of both pathways resulted in reduced contraction and could even attenuate fibrosis^58–62^. Thus, TGF-β influences myofibroblast contraction by enhancing αSMA expression, but not through myosin II expression.

### TGF-β stimulates metabolic pathways to gain stiffer ECM in their microenvironment

Although it has been known that FMT is paired with an increase in glucose uptake, enhanced glycolytic activity and activation of serine synthesis pathway, it is only recently discovered that TGF-β is mainly responsible for these activities. Upon stimulation with TGF-β, fibroblasts showed higher glucose consumption, higher ATP levels and higher oxygen consumption rates. When tracing glucose through the cell, it was found that the higher glucose consumption does not only result in elevated lactate levels but also serine and glycine are produced from the glucose consumption upon TGF-β stimulation^63^. Remarkable, TGF-β also increased the labelling of multiple TCA cycle metabolites from glucose^63^, whereas it has been implied that TGF-β decouples the TCA cycle from the glycolysis to produce more ATP^19,20,64,65^. The uncoupling from oxidative phosphorylation is also supported clinically by a metabolomics study of patients with renal fibrosis where they found fewer TCA cycle intermediates^66^. Nevertheless, this was a serum analysis and therefore might not represent the metabolic environment in fibrotic tissues. Other metabolomics studies performed on patients’ biopsies from idiopathic pulmonary fibrotic lungs^67^ and fibrotic liver^68^ support the results found for the glucose flux towards the TCA cycle. This is in line with the higher availability of glucose for biosynthesis and mitochondrial oxidation upon TGF-β stimulation.

Despite of enhanced glycolytic flux, this is not enough to meet the high metabolic demands of myofibroblasts and increased carbon supply through other pathways is needed to support biosynthetic requirements. For example, studies have shown that glutaminolysis is required for TGF-β-induced FMT^21^ and collagen production^22,23^. Myofibroblasts convert glutamate to proline and a knockdown of pyrroline-5-carboxylate synthase (P5CS), the enzyme responsible for this conversion, attenuated the collagen production induced by TGF-β and even supplementing the medium with extracellular proline did not rescue the collagen production^23,63^. This implies de novo synthesis of proline is vital for collagen production. Thus, TGF-β stimulates both glycolysis and glutaminolysis for its collagen production.

**Fig. 3:**
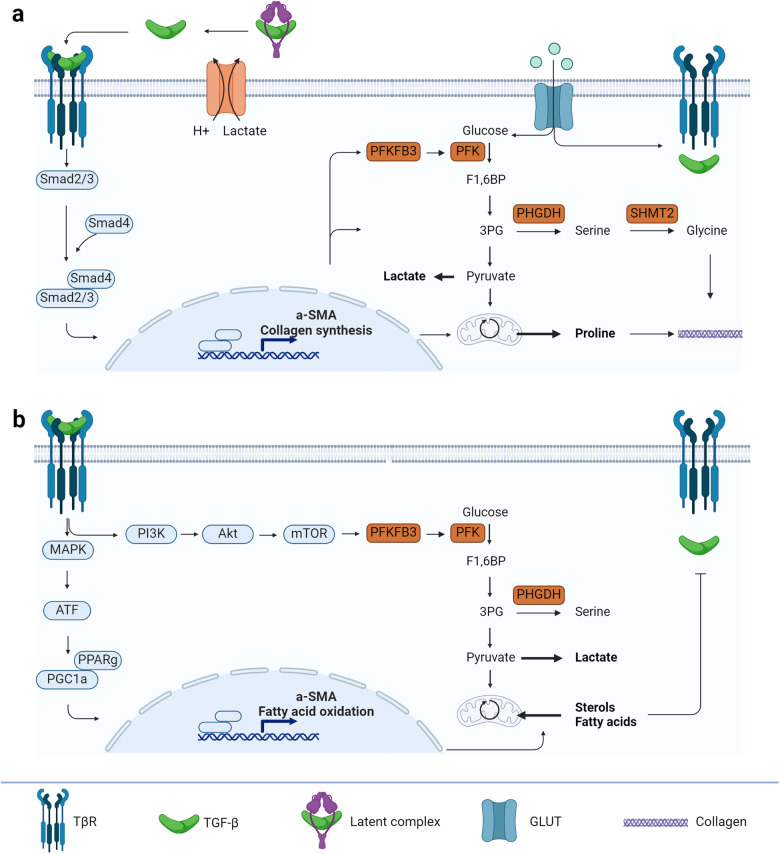
TGF-β influences cellular metabolism through canonical and non-canonical pathways. **a** The Smad complex promotes not only α-smooth muscle actin (αSMA) expression for myofibroblast contraction but also a higher glycolytic rate by increasing the expression of phosphofructo-2-kinase/fructose-2,6-biphosphatase 3 (PFKFB3) and proline synthesis for collagen production. **b** The non-canonical pathway of TGF-β receptors promotes αSMA expression as well as fatty acid oxidation. The decrease in sterols and fatty acid levels in turn inhibits TGF-β and its receptors production. ATF activating transcription factor, GLUT glucose transporter, MAPK mitogen-activated protein kinase, mTOR mammalian target of rapamycin, PFK phosphofructokinase, PGC1a PPAR gamma co-activator-1a, PHGDH phosphoglycerate dehydrogenase, PI3K phosphoinositide 3-kinase, PPARg peroxisome proliferator-activator receptor, SHMT2 serine hydroxymethyltransferase 2. Created with BioRender.com.

The observed higher glucose consumption for elevated lactate, serine, and glycine levels as well as more TCA cycle activity and proline synthesis can either come from the canonical (Fig. 3a) or non-canonical pathway (Fig. 3b). Although clearly aiding FMT, so far, the impact of the canonical signalling pathway on cellular metabolism is understudied. Currently, few studies have investigated the effect of Smad proteins on TGF-β-induced metabolic alterations. For example, a knockdown of Smad2 and Smad3 reduced the TGF-β-induced expression of the rate-limiting enzyme of glycolysis 6-phosphofructo-2-kinase/fructose-2,6-bisphosphatase 3 (PFKFB3)^69^ and the rate-limiting enzyme of serine synthesis pathway phosphoglycerate dehydrogenase (PHGDH) and serine hydroxymethyltransferase 2 (SHMT2)^70^. The last one is responsible for the conversion of serine into glycine^71^, which is known to get incorporated into collagen upon TGF-β stimulation and is a metabolic characteristic for myofibroblasts^23,70,72^. Another hint that Smad proteins are indeed involved in regulating cellular metabolism is the knockdown of Smad4, a common Smad required for nuclear shuttling of R-Smads to act as transcription factors. Smad4-knockdown inhibited directly the TGF-β-induced TCA cycle activity and proline synthesis^21,22,63^. This implies that Smad proteins do influence also FMT by stimulating metabolic alterations. However, more studies must be performed to understand the molecular details.

In contrast to the canonical signalling pathways, the impact of the non-canonical pathway on cellular metabolism has been studied more, mainly the role of mTOR has been identified as the key player in fibrosis. Stimulating human lung fibroblasts with lipopolysaccharide (LPS) activated the PI3K-Akt-mTOR pathway and subsequently caused an upregulation in PFKFB3 expression^73^. Additionally, higher expression levels were found for the serine synthesis pathway due to an accumulation of ATF4 through the PI3K-Akt-mTOR pathway^72^. This further enhanced aerobic glycolysis and promoted collagen production. Moreover, these enhanced expression levels were also detected in the mouse model for LPS-induced pulmonary fibrosis^73^. All these effects and the fibrosis could be almost abolished with the mTOR inhibitor rapamycin, revealing the importance of mTOR in the metabolic reprogramming of fibroblasts into myofibroblasts.

Likewise, MAPK was shown to phosphorylate activating transcription factor 2 (ATF2) in a redox-dependent manner, and consequently induce transcriptional activation of peroxisome proliferator-activator receptor (PPAR) gamma co-activator-1a (PGC-1a)^74^. This stimulates not only glycolysis but also mitochondrial biogenesis which is required for the expression of a-SMA^75^. Additionally, activating PGC-1a is known to cause a mitochondrial calcium uniporter (MCU)-mediated metabolic reprogramming to FAO. Interestingly, a knockdown of MCU in macrophages led to protection against pulmonary fibrosis by promoting glycolysis^76^. This emphasizes the intercellular dependency on the metabolic activities between fibroblasts and immune cells leading to pro-fibrotic conditions. In contrast, there are also studies reporting how MAPK downregulates FAO transcription factors, such as PGC-1a^77,78^. Consequently, an increase in fatty acid synthesis, in TGF-β production and a decrease in ECM degradation could be observed^79^. One explanation of the different mechanisms is that PGC-1a has different possible binding partners. When PGC-1a binds to PPARγ, mitochondrial biosynthesis and FAO is stimulated, whereas binding to PPARα inhibits FAO^77^. This clearly highlights the importance of understanding the molecular details of the involved signalling cascades and their impact on metabolic reprogramming to determine the pathophysiology of fibrosis.

### Energy-producing pathways are important for TGF-β signalling cascades

Extensive evidence indicates that cellular metabolism also influences TGF-β signalling. It started with exposure to high glucose concentrations, which enhanced TGF-β production^80,81^. A surprising aspect of this production is that glucose is not necessarily important. Glucosamine, an intermediate of the hexosamine biosynthetic pathway, was more potent than glucose to enhance the production. Indeed, inhibition of glutamine:fructose-6-phosphate amidotransferase negatively impacted TGF-β production^82^. Nevertheless, high glucose concentrations do stimulate higher TbRI and TbRII levels at the cell membrane^83^. High lactate levels have a similar effect to high glucose concentration^84^ and can be thought of as an effect of high glucose levels. Thus, high glucose concentrations promote TGF-β signalling through enhancing lactate and TGF-β production and more receptors on the cell membrane.

Fatty acids are also important to consider, as they are not only building blocks for cell membranes but are also energy-rich compounds that can be degraded to provide ATP via FAO. As mentioned earlier, this is the pathway that fibroblasts rely on for their homoeostatic state. Fascinatingly, FAO enhanced the expression of TGF-β^85^, whereas its counterpart fatty acid synthesis reduced the TbRI and TbRII levels at the cell membrane. This indicates that ATP production is particularly important to stimulate TGF production and signalling^86^.

In summary, TGF-β promotes the expression of αSMA in fibroblasts which is the key characteristic for myofibroblasts. Intriguingly, the homoeostatic FAO is enough to help in the TGF-β production and stimulate the myofibroblast phenotype. However, TGF-β also stimulates glycolysis and glutaminolysis to promote collagen production and thereby further enhance its autocrine production and secretion, creating a positive feedforward loop. Together, it shows that the energy-producing pathways are important for the working mechanism of TGF-β.

## Connecting TGF-β and integrin signalling through YAP/TAZ

Interestingly, both integrin- and TGF-β signalling are known to be influenced by Yes-associated protein/transcriptional coactivator with PDZ-binding motif (YAP/TAZ), and this can be a common mechanism for regulating cellular metabolism and myofibroblast contraction. YAP and TAZ are paralogous proteins that act as transcription co-activators for the transcriptional enhanced associate domain (TEAD) family of transcription factors to control gene expression^87^. The next subsections will focus on the influence of YAP/TAZ on myofibroblast contraction and its involvement with cellular metabolism.

### Stiff ECM promotes nuclear localisation of YAP/TAZ and causes αSMA transcription in synergy with Smad complex

Myofibroblast contraction regulation by YAP/TAZ is very dependent on its cellular localisation^87^. YAP/TAZ are localised in the cytoplasm in cells that are on soft ECM, whereas stiff ECM promotes YAP/TAZ nuclear localisation through the opening of the nuclear envelope pores by tensioned stress fibres^88,89^. The importance of stress fibres is confirmed by the inhibition of NMMII. The blockage of myosin II decreased the nuclear accumulation of YAP1 and promoted the cytoplasmic localisation of YAP1^87^. By keeping YAP1 in the cytosol, they cannot promote fibrotic gene expression and therefore inhibit fibrosis.

Furthermore, YAP/TAZ is known to influence TGF-β signalling. YAP/TAZ can bind the activated Smad complex in the nucleus and thereby retain the complex in the nucleus to promote αSMA transcription. The binding of YAP/TAZ to the Smad complex, however, only happens on stiff ECM. Soft ECM inhibits TGF-β-induced Smad signalling by controlling Smad2/3 localisation, which is in turn regulated by YAP/TAZ^89,90^. Thus, stiff ECM promotes YAP/TAZ nuclear localisation where it -in synergy with the TGF-β signalling cascade- causes αSMA transcription and influences myofibroblast contraction.

### YAP/TAZ regulates not only glycolysis and glutaminolysis during FMT but also lipid metabolism

**Fig. 4:**
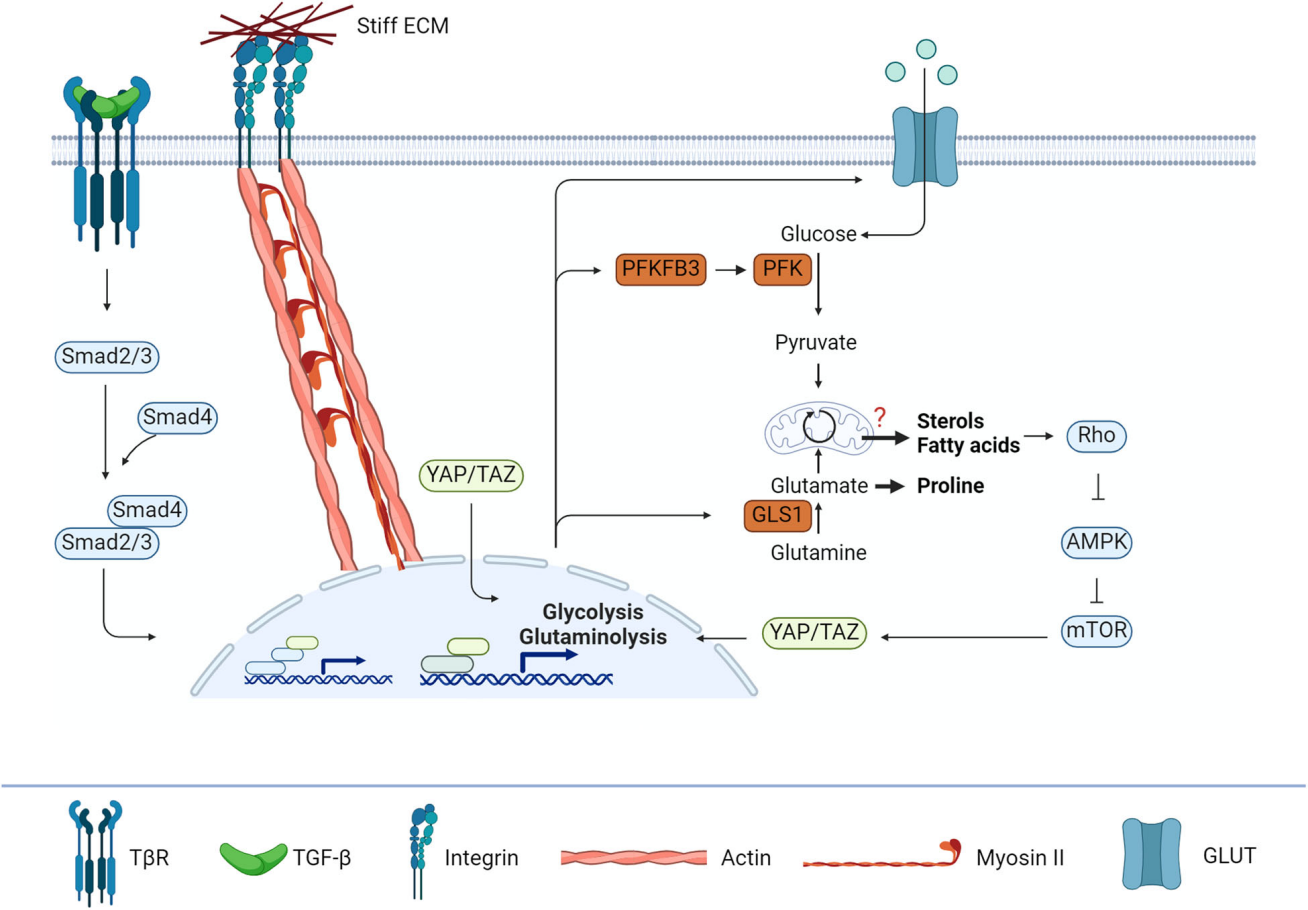
YAP/TAZ bridges the influences of ECM and TGF-β. YAP/TAZ localizes to the nucleus upon stiff ECM where it stabilizes the Smad complex and sustains the gene expression observed for TGF-β. Moreover, YAP/TAZ can also independently promote glycolysis and glutaminolysis by increasing the expression of glucose transporters (GLUTs), of phosphofructo-2-kinase/fructose-2,6-biphosphatase 3 (PFKFB3) and of glutamine synthesis (GLS1) for the collagen production. As a consequence, increased sterol and fatty acid levels promote more Rho expression and thereby promote a feedforward loop. AMPK AMP-activated protein kinase, mTOR mammalian target of rapamycin, PFK phosphofructokinase.

It is known that several metabolic pathways can be regulated by YAP/TAZ (Fig. 4). First, glycolysis gets promoted by YAP/TAZ nuclear localisation. Here, YAP/TAZ stimulate glucose transporter 1 (GLUT1) transcription^91^. Additionally, hexokinase and PFKB3 are induced by YAP/TAZ^92^, which together with GLUT1 expression causes increased glycolytic activity in fibroblasts. Second, YAP/TAZ regulate the expression of glutaminase 1 (GLS1) and phosphoserine aminotransferase (PSAT1)^93^, the enzymes responsible for glutaminolysis. Interestingly, the metabolic reprogramming of glutaminolysis is dependent on YAP-mediated induction of GLS1. This was shown by inhibition of YAP, which led to inhibited glutaminolysis and suppression of fibrosis^94,95^. Moreover, this confirms the earlier observations that glutaminolysis becomes important for FMT.

In addition to the central carbon pathways, lipid metabolism seems to be regulated by YAP/TAZ as well. However, there is some contradicting literature. On one hand, YAP/TAZ was found to decrease lipid deposition^96^. On the other hand, it was shown that YAP/TAZ accelerated lipid accumulation^97^. It should be noted that these studies were performed in different tissues, liver, and adipose tissue respectively, and this indicates a tissue-specific response for YAP/TAZ.

### Nutrient availability regulates YAP/TAZ localisation through AMPK and mTOR in a similar manner as the ECM properties

The influence of cellular metabolism on YAP/TAZ has been known for a while. YAP/TAZ is regulated by AMPK and mTOR upon nutrient availability. For example, AMPK inhibits YAP/TAZ activity upon glucose starvation, whereas mTOR promotes YAP/TAZ nuclear localisation upon high glucose concentrations^98,99^. This can provide a feedback loop in the regeneration stages. The initial haematoma stage does not have a nutrient source as vessels are ruptured^100^. This means that glucose levels are very limited, and no ECM is yet produced. Together, the soft ECM and glucose starvation prevent YAP/TAZ from localising into the nucleus, whereas later stages with stiffer ECM and higher glucose levels stimulate YAP/TAZ activation.

YAP/TAZ is also regulated by lipid metabolism. One of the first to be reported as a link between YAP/TAZ and cellular metabolism was an activated mevalonate pathway^101,102^. The mevalonate pathway is the fatty synthesis pathway, which results in cholesterol, bile acids and steroid hormones. Reducing cholesterol levels by statins resulted in efficient suppression of YAP/TAZ nuclear localisation^102^, clearly showing a link between YAP/TAZ and cellular metabolism. Additionally, multiple studies showed that this inhibition was due to Rho inhibition, linking YAP/TAZ back to integrin signalling^101–103^. Thus, YAP/TAZ might provide a common cascade for cellular metabolism and its influence on contraction.

## Conclusion and outlook

**Fig. 5:**
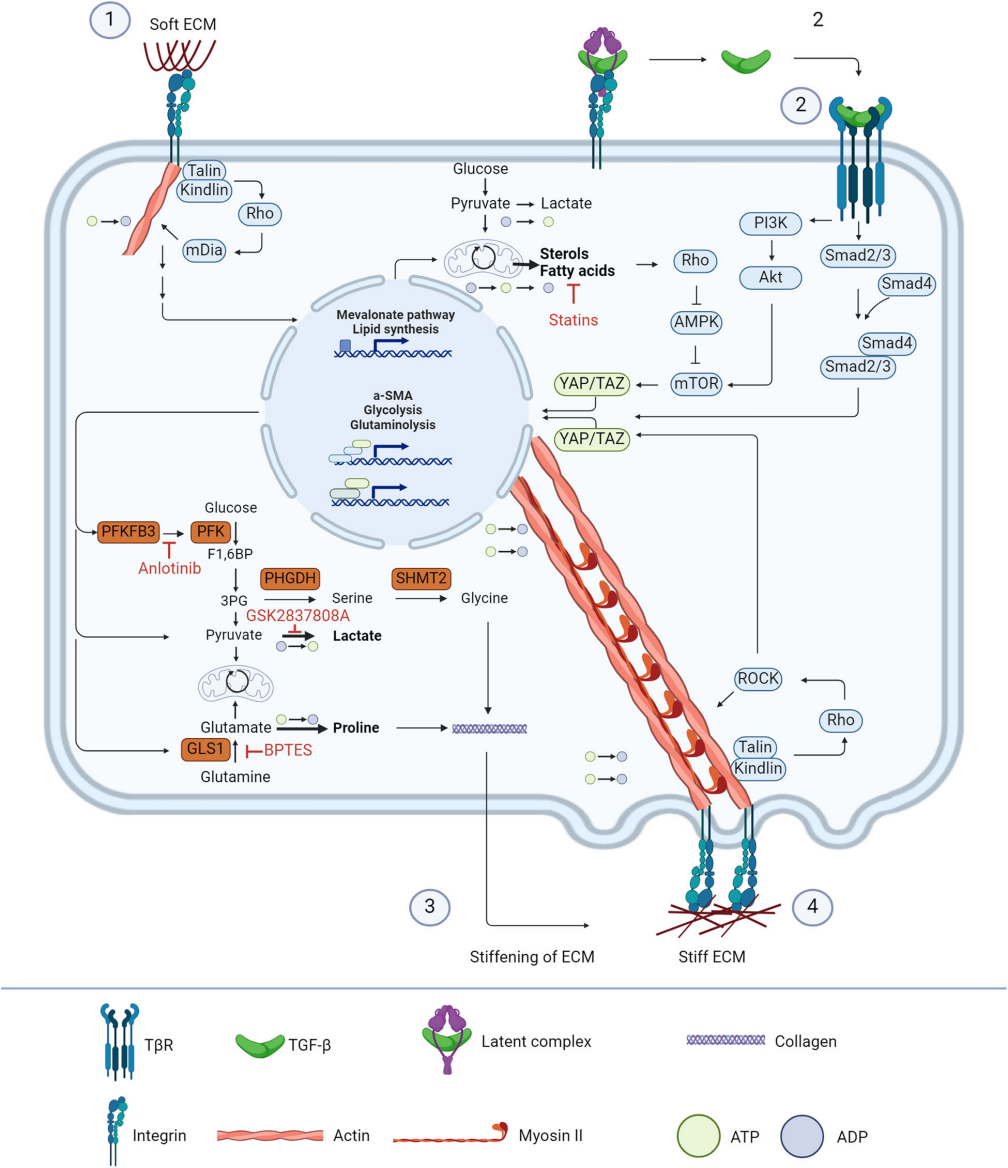
Overview of cellular metabolism and myofibroblast contraction cascades. Soft ECM causes signalling through the Rho-mDia axis, which is often paired with the assembly of actin filaments and upregulation of the mevalonate pathway. Additionally, the same integrin receptors can bind the latency complex of TGF-β thereby causing release of the mature ligand. This, together with YAP/TAZ, then takes over the actin filament synthesis and by increasing the expression of key enzymes in the glycolysis and glutaminolysis the TGF-β and YAP/TAZ axis produce stiffer ECM. Stiff ECM causes the integrin signalling to shift to the Rho-ROCK axis, which is paired with myosin II synthesis and subsequently contraction. Additionally, ROCK promotes YAP/TAZ to be localised in the nucleus to promote a feedforward loop towards stiff ECM and myofibroblast contraction. By inhibiting key points in the feedforward loop, such as the mevalonate pathway with statins^109–112^, glycolysis with Anlotinib^104^, lactate production by GSK2837808A^106^, or glutaminolysis with BPTES^107,108^, it might be possible to break the loop and thereby switch the fibrotic cascades back to regenerative cascades.

A changing metabolic phenotype of myofibroblasts is nowadays more recognised to play a role in collagen production. Myofibroblasts, for instance, have a higher glycolysis and glutaminolysis rate to promote the synthesis of essential amino acids compared to fibroblasts. Nevertheless, the role of cellular metabolism in myofibroblast contraction remained unexplored. This review aimed to provide an overview of the influence of cellular metabolism on myofibroblast contraction (Fig. 5).

We conclude that integrin receptors and TGF-β have similar effects on cellular metabolism and conversely, cellular metabolism has similar effects on integrin receptor and TGF-β signalling. Both receptors enhance glycolysis and inhibit FAO^32,36^, creating a feed-forward loop for better integrin and TGF-β signalling cascades^45,48,80^. Additionally, TGF-β enhances the glutaminolysis activity of myofibroblasts^21,23^, which can also be observed by YAP/TAZ stimulation^94^. It is even suggested that YAP/TAZ is needed for the enhanced glutaminolysis activity since they enhance the transcription of GLS1, the enzyme responsible for the conversion of glutamine to glutamate. This implies that the central carbon metabolic pathways are important for myofibroblast contraction and inhibition might result in attenuated fibrosis. Indeed, glycolysis inhibition alleviated cardiac fibrosis^104^ and joint osteoarthritis^105,106^. GLS inhibition in an airway specimen inhibited scar fibroblast proliferation and function and targeting the glutaminolysis in heart tissue has been shown to reverse fibrosis^107,108^, demonstrating the critical role of GLS and glutaminolysis as well. Together, this shows a metabolic dependency of myofibroblasts during contraction and FMT and could be transferred to a new treatment strategy.

Another promising metabolic pathway that has not yet been researched in detail is the mevalonate pathway. Cholesterol seems to play an important role in regulating responses. It does not only regulate membrane fluidity, hence integrin signalling but also causes reduced TβRI and TβRII levels at the cell membrane and YAP/TAZ in the nucleus. Statins, which are known to not only reduce cholesterol levels but also have anti-inflammatory effects, have been tested in lung fibrosis and showed a decrease in fibrotic tissue^109,110^. Similarly, cardiac and liver fibrosis improved by statin treatment^111,112^. However, it is worth mentioning that depletion of cholesterol does result in enhanced TGF-β sensitivity due to increased Smad2/3 expression and phosphorylation^113^. Despite of the higher TGF-β sensitivity, reduced cholesterol levels might still cause a less lasting effect on the Smad proteins since YAP/TAZ nuclear localisation is reduced and therefore statins might still be effective. Hence, the mevalonate pathway might also form a solution for better effective drugs in clinical use after fibrotic tissue has occurred.

Despite these promising perspectives, several questions remain: Most studies reported here focused on the effect of mechanics on metabolism and did not consider cellular contraction. As metabolism is known to modulate a lot of cellular processes, it should be interesting to look at how metabolism is also altering the mechano-transduction and contraction of cells. Additionally, most studies analysed in this review have been performed in 2D in vitro or ex vivo experiments. This does not take the microenvironment of the cells into account as they would have experienced in vivo. A recent development in cancer research is the use of 3D spheroids that have revealed 3D-specific behaviours^114,115^. As this is also of importance for tissue regeneration as described in our review, we propose to develop and implement 3D tissue engineering techniques for tissue fibrosis that allow for metabolic analysis. By combining the 3D environment with biochemical signals, such as TGF-β, and mechanical stimuli, such as changing biophysical properties of the ECM, a better understanding can be reached of the conditions that cells experience in tissues and organs in vivo. Moreover, different tissues have different tissue stiffness, different fibroblast subsets and different resident metabolites and therefore different homoeostatic metabolic states as well as homoeostatic cytokine states. This can massively alter the response of the fibroblasts used for a study. Hence, by implementing the tissue-specific stiffness, fibroblast subsets and homoeostatic cytokine and metabolic states in the 3D tissue environments, a cross-examining of tissues and organs can be performed to assess whether there is a common mechanism between cellular metabolism and myofibroblast contraction. Only then, effective drugs can be designed for clinical use.

## References

[1] Wynn TA (2008). Cellular and molecular mechanisms of fibrosis. J. Pathol..

[2] Gomes RN, Manuel F, Nascimento DS (2021). The bright side of fibroblasts: molecular signature and regenerative cues in major organs. npj Regen. Med..

[3] Friedman SL, Sheppard D, Duffield JS, Violette S (2013). Therapy for fibrotic diseases: Nearing the starting line. Sci. Transl. Med..

[4] Wynn, T. A. Fibrotic disease and the TH1/TH2 paradigm. *Nat. Rev. Immunol.* **4**, 583–594 (2004). 10.1038/nri1412 PMC2702150 15286725

[5] Walker M, Godin M, Pelling AE (2020). Mechanical stretch sustains myofibroblast phenotype and function in microtissues through latent TGF-β1 activation. Integr. Biol..

[6] D’Urso M, Kurniawan NA (2020). Mechanical and Physical Regulation of Fibroblast–Myofibroblast Transition: From Cellular Mechanoresponse to Tissue Pathology. Front Bioeng. Biotechnol..

[7] He Z-Q (2022). Pharmacological regulation of tissue fibrosis by targeting the mechanical contraction of myofibroblasts. Fundam. Res..

[8] Annes JP, Rifkin DB, Munger JS (2002). The integrin α _V_ β _6_ binds and activates latent TGFβ3. FEBS Lett..

[9] Asano Y, Ihn H, Yamane K, Jinnin M, Tamaki K (2006). Increased Expression of Integrin αvβ5 Induces the Myofibroblastic Differentiation of Dermal Fibroblasts. Am. J. Pathol..

[10] Denton CP (2007). Recombinant human anti–transforming growth factor β1 antibody therapy in systemic sclerosis: A multicenter, randomized, placebo-controlled phase I/II trial of CAT-192. Arthritis Rheum..

[11] Douglass A (2008). Antibody-targeted myofibroblast apoptosis reduces fibrosis during sustained liver injury. J. Hepatol..

[12] Teixeira AF, ten Dijke P, Zhu HJ (2020). On-Target Anti-TGF-β Therapies Are Not Succeeding in Clinical Cancer Treatments: What Are Remaining Challenges?. Front. Cell Dev. Biol..

[13] Brauer, E. et al. Collagen Fibrils Mechanically Contribute to Tissue Contraction in an In Vitro Wound Healing Scenario. *Adv. Sci.*10.1002/advs.201801780 (2019). 10.1002/advs.201801780 PMC6498124 31065517

[14] Vallée A, Lecarpentier Y (2019). TGF-β in fibrosis by acting as a conductor for contractile properties of myofibroblasts. Cell Biosci..

[15] Zaal EA, Berkers CR (2018). The Influence of Metabolism on Drug Response in Cancer. Front. Oncol..

[16] Rizzieri D, Paul B, Kang Y (2019). Metabolic alterations and the potential for targeting metabolic pathways in the treatment of multiple myeloma. J. Cancer Metastasis Treat..

[17] Maes K, Menu E (2018). Metabolic Features of Multiple Myeloma. Int J. Mol. Sci..

[18] Kim S-Y (2018). Cancer Energy Metabolism: Shutting Power off Cancer Factory. Biomol. Ther..

[19] Smith ER, Hewitson TD (2020). TGF-β1 is a regulator of the pyruvate dehydrogenase complex in fibroblasts. Sci. Rep..

[20] Ding H (2017). Inhibiting aerobic glycolysis suppresses renal interstitial fibroblast activation and renal fibrosis. Am. J. Physiol. Ren. Physiol..

[21] Bernard K (2018). Glutaminolysis is required for transforming growth factor-β1–induced myofibroblast differentiation and activation. J. Biol. Chem..

[22] Ge J (2018). Glutaminolysis Promotes Collagen Translation and Stability via α-Ketoglutarate–mediated mTOR Activation and Proline Hydroxylation. Am. J. Respir. Cell Mol. Biol..

[23] Hamanaka, R. B. et al. Glutamine Metabolism Is Required for Collagen Protein Synthesis in Lung Fibroblasts. *Am. J. Respir. Cell Mol. Biol.*10.1165/rcmb.2019-0008OC (2019). 10.1165/rcmb.2019-0008OC PMC6827066 30973753

[24] Vander Heiden MG (2011). Targeting cancer metabolism: a therapeutic window opens. Nat. Rev. Drug Discov..

[25] Romani, P., Valcarcel-Jimenez, L., Frezza, C. & Dupont, S. Crosstalk between mechanotransduction and metabolism. *Nat. Rev. Mol. Cell Biol.*10.1038/s41580-020-00306-w. 10.1038/s41580-020-00306-w 33188273

[26] Theodosiou M (2016). Kindlin-2 cooperates with talin to activate integrins and induces cell spreading by directly binding paxillin. Elife.

[27] Kanchanawong P (2010). Nanoscale architecture of integrin-based cell adhesions. Nature.

[28] Leahy DJ, Hendrickson WA, Aukhil l, Erickson HP (1992). Structure of a Fibronectin Type III Domain from Tenascin Phased by MAD Analysis of the Selenomethionyl Protein. Science.

[29] Wong SW, Lenzini S, Cooper MH, Mooney DJ, Shin J-W (2020). Soft extracellular matrix enhances inflammatory activation of mesenchymal stromal cells to induce monocyte production and trafficking. Sci. Adv..

[30] Narumiya S, Tanji M, Ishizaki T (2009). Rho signaling, ROCK and mDia1, in transformation, metastasis and invasion. Cancer Metastasis Rev..

[31] Lu Y-Y (2020). Nonmuscle Myosin II Activation Regulates Cell Proliferation, Cell Contraction, and Myofibroblast Differentiation in Keloid-Derived Fibroblasts. Adv. Wound Care.

[32] Romani P (2019). Extracellular matrix mechanical cues regulate lipid metabolism through Lipin-1 and SREBP. Nat. Cell Biol..

[33] Bertolio R (2019). Sterol regulatory element binding protein 1 couples mechanical cues and lipid metabolism. Nat. Commun..

[34] Singh, V., Erady, C. & Balasubramanian, N. Cell-matrix adhesion controls Golgi organization and function through Arf1 activation in anchorage-dependent cells. *J. Cell Sci.*10.1242/jcs.215855 (2018). 10.1242/jcs.215855 PMC6127727 30054383

[35] Zhao X, Yang F (2012). Regulation of SREBP-Mediated Gene Expression. Acta Biophys. Sin..

[36] Park JS (2020). Mechanical regulation of glycolysis via cytoskeleton architecture. Nature.

[37] Noda K (2014). Rho-Kinase Inhibition Ameliorates Metabolic Disorders through Activation of AMPK Pathway in Mice. PLoS One.

[38] Tominaga T (1998). p160ROCK mediates RhoA activation of Na-H exchange. EMBO J..

[39] Tominaga T, Barber DL (1998). Na–H Exchange Acts Downstream of RhoA to Regulate Integrin-induced Cell Adhesion and Spreading. Mol. Biol. Cell.

[40] Wang Y (2020). Drp1-mediated mitochondrial fission promotes renal fibroblast activation and fibrogenesis. Cell Death Dis..

[41] Hasan P (2018). Mitochondrial fission protein, dynamin-related protein 1, contributes to the promotion of hypertensive cardiac hypertrophy and fibrosis in Dahl-salt sensitive rats. J. Mol. Cell Cardiol..

[42] Steffen J (2022). The mitochondrial fission protein Drp1 in liver is required to mitigate NASH and prevents the activation of the mitochondrial ISR. Mol. Metab..

[43] Zanotelli MR (2018). Regulation of ATP utilization during metastatic cell migration by collagen architecture. Mol. Biol. Cell.

[44] Kusakabe T, Motoki K, Hori K (1997). Mode of Interactions of Human Aldolase Isozymes with Cytoskeletons. Arch. Biochem. Biophys..

[45] Wang J, Morris AJ, Tolan DR, Pagliaro L (1996). The Molecular Nature of the F-actin Binding Activity of Aldolase Revealed with Site-directed Mutants. J. Biol. Chem..

[46] Taylor KA, Taylor DW (1994). Formation of two-dimensional complexes of F-actin and crosslinking proteins on lipid monolayers: demonstration of unipolar alpha-actinin-F-actin crosslinking. Biophys. J..

[47] Hąc-Wydro K, Wydro P (2007). The influence of fatty acids on model cholesterol/phospholipid membranes. Chem. Phys. Lipids.

[48] Gopalakrishna P, Chaubey SK, Manogaran PS, Pande G (2000). Modulation of alpha5beta1 integrin functions by the phospholipid and cholesterol contents of cell membranes. J. Cell Biochem..

[49] Pokharel SM (2019). Integrin activation by the lipid molecule 25-hydroxycholesterol induces a proinflammatory response. Nat. Commun..

[50] Boulter E (2018). Cell metabolism regulates integrin mechanosensing via an SLC3A2-dependent sphingolipid biosynthesis pathway. Nat. Commun..

[51] Hua W, ten Dijke P, Kostidis S, Giera M, Hornsveld M (2020). TGFβ-induced metabolic reprogramming during epithelial-to-mesenchymal transition in cancer. Cell. Mol. Life Sci..

[52] Smoktunowicz N (2015). The anti-fibrotic effect of inhibition of TGFβ-ALK5 signalling in experimental pulmonary fibrosis in mice is attenuated in the presence of concurrent γ-herpesvirus infection. Dis. Model Mech..

[53] Meng X, Nikolic-Paterson DJ, Lan HY (2016). TGF-β: the master regulator of fibrosis. Nat. Rev. Nephrol..

[54] Zhao M (2022). Targeting fibrosis: mechanisms and clinical trials. Signal Transduct. Target Ther..

[55] Fernandez IE, Eickelberg O (2012). The Impact of TGF-β on Lung Fibrosis. Proc. Am. Thorac. Soc..

[56] Saadat S (2021). Pivotal Role of TGF-β/Smad Signaling in Cardiac Fibrosis: Non-coding RNAs as Effectual Players. Front. Cardiovasc. Med..

[57] Hu H-H (2018). New insights into TGF-β/Smad signaling in tissue fibrosis. Chem. Biol. Interact..

[58] Abdalla M, Goc A, Segar L, Somanath PR (2013). Akt1 Mediates α-Smooth Muscle Actin Expression and Myofibroblast Differentiation via Myocardin and Serum Response Factor. J. Biol. Chem..

[59] Hanna A, Humeres C, Frangogiannis NG (2021). The role of Smad signaling cascades in cardiac fibrosis. Cell Signal.

[60] Hui ST (2021). Oxy210, a novel inhibitor of hedgehog and TGF‐β signalling, ameliorates hepatic fibrosis and hypercholesterolemia in mice. Endocrinol. Diabetes Metab..

[61] Gwon M-G (2021). Apamin inhibits renal fibrosis via suppressing TGF-β1 and STAT3 signaling in vivo and in vitro. J. Mol. Med..

[62] Liu Q (2016). Salvianolic Acid B Attenuates Experimental Pulmonary Fibrosis through Inhibition of the TGF-β Signaling Pathway. Sci. Rep..

[63] Schwörer S (2020). Proline biosynthesis is a vent for TGFβ‐induced mitochondrial redox stress. EMBO J..

[64] Hewitson TD, Smith ER (2021). A Metabolic Reprogramming of Glycolysis and Glutamine Metabolism Is a Requisite for Renal Fibrogenesis—Why and How?. Front. Physiol..

[65] Smith ER, Wigg B, Holt SG, Hewitson TD (2019). TGF-β1 modifies histone acetylation and acetyl-coenzyme A metabolism in renal myofibroblasts. Am. J. Physiol. Ren. Physiol..

[66] Kwan B (2020). Metabolomic Markers of Kidney Function Decline in Patients With Diabetes: Evidence From the Chronic Renal Insufficiency Cohort (CRIC) Study. Am. J. Kidney Dis..

[67] Zhao YD (2017). Metabolic heterogeneity of idiopathic pulmonary fibrosis: a metabolomic study. BMJ Open Respir. Res..

[68] He X (2022). Metabolomic Profiling for Histologically Fibrotic Stage in Chronic Drug-Induced Liver Injury. Front. Pharm..

[69] Xie N (2015). Glycolytic Reprogramming in Myofibroblast Differentiation and Lung Fibrosis. Am. J. Respir. Crit. Care Med..

[70] Nigdelioglu R (2016). Transforming Growth Factor (TGF)-β Promotes de Novo Serine Synthesis for Collagen Production. J. Biol. Chem..

[71] Rathore, R., Schutt, C. R. & Van Tine, B. A. PHGDH as a mechanism for resistance in metabolically-driven cancers. *Cancer Drug Resist.*10.20517/cdr.2020.46 (2020). 10.20517/cdr.2020.46 PMC7840151 33511334

[72] O’Leary EM (2020). TGF-β Promotes Metabolic Reprogramming in Lung Fibroblasts via mTORC1-dependent ATF4 Activation. Am. J. Respir. Cell Mol. Biol..

[73] Hu X (2020). PI3K-Akt-mTOR/PFKFB3 pathway mediated lung fibroblast aerobic glycolysis and collagen synthesis in lipopolysaccharide-induced pulmonary fibrosis. Lab. Investig..

[74] Cao W (2004). p38 Mitogen-Activated Protein Kinase Is the Central Regulator of Cyclic AMP-Dependent Transcription of the Brown Fat Uncoupling Protein 1 Gene. Mol. Cell Biol..

[75] Bernard K (2015). Metabolic Reprogramming Is Required for Myofibroblast Contractility and Differentiation. J. Biol. Chem..

[76] Gu L (2019). Mitochondrial calcium uniporter regulates PGC-1α expression to mediate metabolic reprogramming in pulmonary fibrosis. Redox Biol..

[77] Zhang J (2020). Molecular Profiling Reveals a Common Metabolic Signature of Tissue Fibrosis. Cell Rep. Med..

[78] Palomer X (2009). TNF-α reduces PGC-1α expression through NF-κB and p38 MAPK leading to increased glucose oxidation in a human cardiac cell model. Cardiovasc. Res..

[79] Zhu X (2021). Metabolic Reprogramming and Renal Fibrosis. Front. Med..

[80] Ziyadeh FN, Sharma K, Ericksen M, Wolf G (1994). Stimulation of collagen gene expression and protein synthesis in murine mesangial cells by high glucose is mediated by autocrine activation of transforming growth factor-beta. J. Clin. Investig..

[81] Kolm V, Sauer U, Olgemooller B, Schleicher ED (1996). High glucose-induced TGF-beta 1 regulates mesangial production of heparan sulfate proteoglycan. Am. J. Physiol. Ren. Physiol..

[82] Kolm-Litty V, Sauer U, Nerlich A, Lehmann R, Schleicher ED (1998). High glucose-induced transforming growth factor beta1 production is mediated by the hexosamine pathway in porcine glomerular mesangial cells. J. Clin. Investig..

[83] Wu L, Derynck R (2009). Essential Role of TGF-β Signaling in Glucose-Induced Cell Hypertrophy. Dev. Cell.

[84] Takahashi H (2019). TGF-β2 is an exercise-induced adipokine that regulates glucose and fatty acid metabolism. Nat. Metab..

[85] Guh J-Y (2003). β-hydroxybutyrate–induced growth inhibition and collagen production in HK-2 cells are dependent on TGF-β and Smad3. Kidney Int..

[86] Gencer, S. et al. TGF-β receptor I/II trafficking and signaling at primary cilia are inhibited by ceramide to attenuate cell migration and tumor metastasis. *Sci Signal* **10**, eaam7464 (2017). 10.1126/scisignal.aam7464 PMC5818989 29066540

[87] Dupont S (2011). Role of YAP/TAZ in mechanotransduction. Nature.

[88] Maître J-L (2016). Asymmetric division of contractile domains couples cell positioning and fate specification. Nature.

[89] Fischer M, Rikeit P, Knaus P, Coirault C (2016). YAP-Mediated Mechanotransduction in Skeletal Muscle. Front. Physiol..

[90] Szeto SG (2016). YAP/TAZ Are Mechanoregulators of TGF- *β* -Smad Signaling and Renal Fibrogenesis. J. Am. Soc. Nephrol..

[91] Cox AG (2018). Yap regulates glucose utilization and sustains nucleotide synthesis to enable organ growth. EMBO J..

[92] Zheng X (2017). LncRNA wires up Hippo and Hedgehog signaling to reprogramme glucose metabolism. EMBO J..

[93] Yang C (2018). Glutamine‐utilizing transaminases are a metabolic vulnerability of TAZ/YAP‐activated cancer cells. EMBO Rep..

[94] Cox AG (2016). Yap reprograms glutamine metabolism to increase nucleotide biosynthesis and enable liver growth. Nat. Cell Biol..

[95] Du K (2018). Hedgehog-YAP Signaling Pathway Regulates Glutaminolysis to Control Activation of Hepatic Stellate Cells. Gastroenterology.

[96] Tharp KM (2018). Actomyosin-Mediated Tension Orchestrates Uncoupled Respiration in Adipose Tissues. Cell Metab..

[97] Aylon Y (2016). The LATS2 tumor suppressor inhibits SREBP and suppresses hepatic cholesterol accumulation. Genes Dev..

[98] Mo J-S (2015). Cellular energy stress induces AMPK-mediated regulation of YAP and the Hippo pathway. Nat. Cell Biol..

[99] Wang W (2015). AMPK modulates Hippo pathway activity to regulate energy homeostasis. Nat. Cell Biol..

[100] Loeffler, J., Duda, G. N., Sass, F. A. & Dienelt, A. The Metabolic Microenvironment Steers Bone Tissue Regeneration. *Trends Endocrinol. Metabol.* **29**, 99–110 (2018). 10.1016/j.tem.2017.11.008 29290501

[101] Wang, Z. et al. Interplay of mevalonate and Hippo pathways regulates RHAMM transcription via YAP to modulate breast cancer cell motility. *Proc. Natl Acad. Sci.* **111**, E89–98 (2014). 10.1073/pnas.1319190110 PMC3890879 24367099

[102] Sorrentino G (2014). Metabolic control of YAP and TAZ by the mevalonate pathway. Nat. Cell Biol..

[103] Mi W (2015). Geranylgeranylation signals to the Hippo pathway for breast cancer cell proliferation and migration. Oncogene.

[104] Chen W (2021). Anlotinib Inhibits PFKFB3-Driven Glycolysis in Myofibroblasts to Reverse Pulmonary Fibrosis. Front. Pharm..

[105] Chen Z-T (2021). Glycolysis Inhibition Alleviates Cardiac Fibrosis After Myocardial Infarction by Suppressing Cardiac Fibroblast Activation. Front. Cardiovasc. Med..

[106] Li HM (2020). Inhibition of glycolysis by targeting lactate dehydrogenase A facilitates hyaluronan synthase 2 synthesis in synovial fibroblasts of temporomandibular joint osteoarthritis. Bone.

[107] Tsai H (2020). Inhibition of glutaminase to reverse fibrosis in iatrogenic laryngotracheal stenosis. Laryngoscope.

[108] Yoshikawa S (2022). Inhibition of glutaminase 1-mediated glutaminolysis improves pathological cardiac remodeling. Am. J. Physiol. Heart Circulatory Physiol..

[109] Santos DM (2020). Screening for YAP Inhibitors Identifies Statins as Modulators of Fibrosis. Am. J. Respir. Cell Mol. Biol..

[110] Michalik M (2013). Lovastatin-induced decrease of intracellular cholesterol level attenuates fibroblast-to-myofibroblast transition in bronchial fibroblasts derived from asthmatic patients. Eur. J. Pharm..

[111] deFilippi C (2018). Brief Report: Statin Effects on Myocardial Fibrosis Markers in People Living With HIV. JAIDS J. Acquired Immune Defic. Syndromes.

[112] Janicko M, Drazilova S, Pella D, Fedacko J, Jarcuska P (2016). Pleiotropic effects of statins in the diseases of the liver. World J. Gastroenterol..

[113] Shapira KE, Ehrlich M, Henis YI (2018). Cholesterol depletion enhances TGF-β Smad signaling by increasing c-Jun expression through a PKR-dependent mechanism. Mol. Biol. Cell.

[114] Muir, A., Danai, L. V. & Vander Heiden, M. G. Microenvironmental regulation of cancer cell metabolism: implications for experimental design and translational studies. *Dis. Model Mech.* **11**, dmm035758 (2018). 10.1242/dmm.035758 PMC6124553 30104199

[115] DelNero P, Hopkins BD, Cantley LC, Fischbach C (2018). Cancer metabolism gets physical. Sci. Transl. Med..

